# Nociceptor-immune interactomes reveal insult-specific immune signatures of pain

**DOI:** 10.1038/s41590-024-01857-2

**Published:** 2024-05-28

**Authors:** Aakanksha Jain, Benjamin M. Gyori, Sara Hakim, Ashish Jain, Liang Sun, Veselina Petrova, Shamsuddin A. Bhuiyan, Shannon Zhen, Qing Wang, Riki Kawaguchi, Samuel Bunga, Daniel G. Taub, M. Carmen Ruiz-Cantero, Candace Tong-Li, Nicholas Andrews, Masakazu Kotoda, William Renthal, Peter K. Sorger, Clifford J. Woolf

**Affiliations:** 1 F. M. Kirby Neurobiology Center, Boston Children’s Hospital, Boston, MA USA; 2 Laboratory of Systems Pharmacology, Harvard Medical School, Boston, MA USA; 3 Khoury College of Computer Sciences, Northeastern University, Boston, MA USA; 4 Department of Bioengineering, College of Engineering, Northeastern University, Boston, MA USA; 5 Department of Neurobiology, Harvard Medical School, Boston, MA USA; 6 Research Computing, Department of Information Technology, Boston Children’s Hospital, Boston, MA USA; 7 Department of Neurology, Brigham and Women’s Hospital and Harvard Medical School, Boston, MA USA; 8 Program in Neurogenetics, Department of Neurology, David Geffen School of Medicine, University of California Los Angeles, Los Angeles, CA USA; 9 Center for Neurobehavioral Genetics, Semel Institute for Neuroscience and Human Behavior, University of California Los Angeles, Los Angeles, CA USA; 10 Department of Pharmacology and Neurosciences Institute (Biomedical Research Center) and Biosanitary Research Institute ibs.GRANADA, University of Granada, Granada, Spain; 11 Salk Institute for Biological Studies, La Jolla, CA USA; 12 Department of Anesthesiology, Faculty of Medicine, University of Yamanashi, Chuo, Yamanashi, Japan; 13 Department of Systems Biology, Harvard Medical School, Boston, MA USA

**Keywords:** Neuroimmunology, Inflammation

## Abstract

Inflammatory pain results from the heightened sensitivity and reduced threshold of nociceptor sensory neurons due to exposure to inflammatory mediators. However, the cellular and transcriptional diversity of immune cell and sensory neuron types makes it challenging to decipher the immune mechanisms underlying pain. Here we used single-cell transcriptomics to determine the immune gene signatures associated with pain development in three skin inflammatory pain models in mice: zymosan injection, skin incision and ultraviolet burn. We found that macrophage and neutrophil recruitment closely mirrored the kinetics of pain development and identified cell-type-specific transcriptional programs associated with pain and its resolution. Using a comprehensive list of potential interactions mediated by receptors, ligands, ion channels and metabolites to generate injury-specific neuroimmune interactomes, we also uncovered that thrombospondin-1 upregulated by immune cells upon injury inhibited nociceptor sensitization. This study lays the groundwork for identifying the neuroimmune axes that modulate pain in diverse disease contexts.

## Abstract (web summary)

Woolf and colleagues use single-cell transcriptomics to determine the gene signature of infiltrating immune cells and potential cell–cell interactions between receptors, ligands, ion channels and metabolites expressed on immune cells and sensory neurons in three models of pain.

## Main

Inflammatory pain is associated with autoimmune diseases, tissue injury and infections. Under healthy circumstances, pain is protective as it is triggered only when specialized sensory neurons, called nociceptors, sense and respond to damaging stimuli such as noxious heat, chemicals or high mechanical force^1^. However, during tissue inflammation, inflammatory mediators released from immune cells act on nociceptors to reduce their threshold for activation, resulting in peripheral sensitization, a major driver of pain at the site of inflammation^1^. Since this is an immune stimulus-triggered process, immune ligands are an attractive target to alleviate inflammatory pain hypersensitivity. Indeed, nonsteroidal anti-inflammatory drugs are effective against inflammatory pain by inhibiting prostaglandin production; however, chronic nonsteroidal anti-inflammatory drug use can cause serious side effects such as gastric lining corrosion and blood pressure dysregulation, necessitating the identification of new neuroimmune targets to treat pain^2^. However, due to the large variety and complex temporal dynamics of immune cells in inflamed tissues, our understanding of the immune mechanisms governing peripheral sensitization remains limited. The quality of the immune response is also dictated by the type of insult^3^. Additionally, sensory neurons in the dorsal root ganglia (DRG) are a heterogeneous population that could react differently to different immune ligands^4^. The resultant inflammatory pain hypersensitivity is, therefore, a cumulative outcome of a large array of ligands produced by various immune cells acting on distinct neuronal populations in an injury-specific manner^5^. In this Resource, to comprehensively map the neuroimmune landscape of inflammatory pain, we decided to characterize how the immune population changes within painful, inflamed tissues in diverse inflammatory pain conditions. We performed a single-cell transcriptomic analysis (single-cell RNA sequencing, scRNA-seq) of skin immune cells at 4, 24 and 48 h following inflammatory insults with zymosan injection, skin incision and ultraviolet (UV) burn to represent distinct clinical conditions: pathogen invasion, trauma and burn injury. We complemented the immune transcriptomics with a single-nucleus RNA sequencing (snRNA-seq) dataset of DRG neurons using a receptor–ligand database compiled by an automated knowledge assembly tool to annotate pain-related neuroimmune axes^6^. This approach revealed neuroimmune interactions specific to particular inflammatory pain conditions, as well as immune regulators of pain, conserved across diverse injury types and with, therefore, broader therapeutic potential.

## Results

### Kinetics of immune infiltration correlate with pain development

**Fig. 1:**
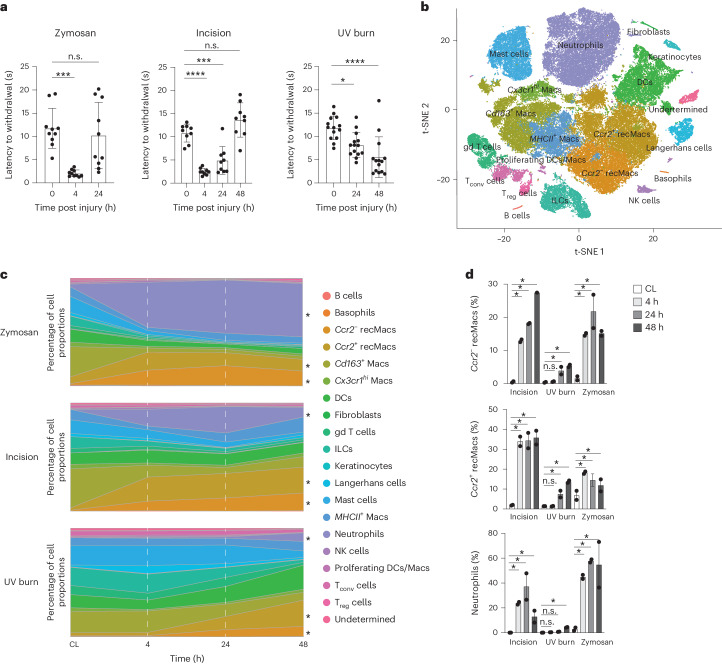
Kinetics of immune infiltration correlate with pain development. **a**, Heat hypersensitivity in inflamed paws measured by the latency to react in the Hargreaves assay before and 4, 24 and 48 h after zymosan injection (*n* = 9, male 4, female 5), incision (*n* = 9, male 4, female 5), UV burn (*n* = 14, male 5, female 9) in the paws of wild-type (WT) mice. Data are represented as mean value ± s.e.m. *P* values calculated using one-way ANOVA, Tukey’s multiple comparison test; 8–12-week-old mice were used. **b**, t-SNE plot of scRNA-seq data of hematopoietic CD45^+^ cells-enriched skin from WT mice integrated from all samples. This comprises zymosan injection, incision and UV burn at 4 h, 24 h and 48 h post-injury and control skin from the CL paws at 4 h (zymosan), 24 h (incision) and 48 h (UV burn). **c**, Stacked area plot of mean proportions of immune cell types at 4 h, 24 h and 48 h in zymosan injection, incision and UV burn and CL healthy skin as in **b**. *Ccr2*^−^ recMacs, *Ccr2*^*+*^ recMacs and neutrophils were significantly changed following injury and are marked with an asterisk. **d**, Proportions of *Ccr2*^−^ recMacs, *Ccr2*^*+*^ recMacs and neutrophils at 4 h, 24 h and 48 h in zymosan, incision and UV burn injury. *Significant change in cell proportions compared to CL based on scCODA analysis. n.s., no statistically significant change in proportions of the cell type based on scCODA analysis (Methods).

Hind paw of 8–12-week-old C57BL/6J wild-type mice were subcutaneously injected with 20 μl of 5 mg ml^−1^ zymosan in saline (hereafter zymosan model), subjected to 3 mm incision on the plantar surface of the hind paw using a sterile scalpel followed by two to three sutures (hereafter incision model), or exposed to UV irradiation at an intensity of 1 J cm^−2^ for 2 min (hereafter UV burn model). Pain hypersensitivity was assessed using the Hargreaves assay, which consists of evaluating the time taken to withdraw the injured paw upon exposure to a heat source, at 4, 24 and 48 h post-injury. Mice subjected to zymosan or skin incision showed a significant heat hypersensitivity within 4 h compared to the pre-injury baseline, which resolved by 24 and 48 h, respectively (Fig. 1a), while significant heat hypersensitivity in the UV burn mice developed later, at 24 h, and was still high at 48 h (Fig. 1a). The time point of peak of hypersensitivity is hereafter referred to as *T*_max_, which is 4 h for zymosan (*T*_max,Zy_), 24 h for incision (*T*_max,In_) and 48 h for UV burn (*T*_max,UV_) injury. The temporal dynamics of hypersensitivity onset and recovery in these inflammatory pain conditions suggested that distinct immune responses might be involved.

**Extended Data Fig. 1:**
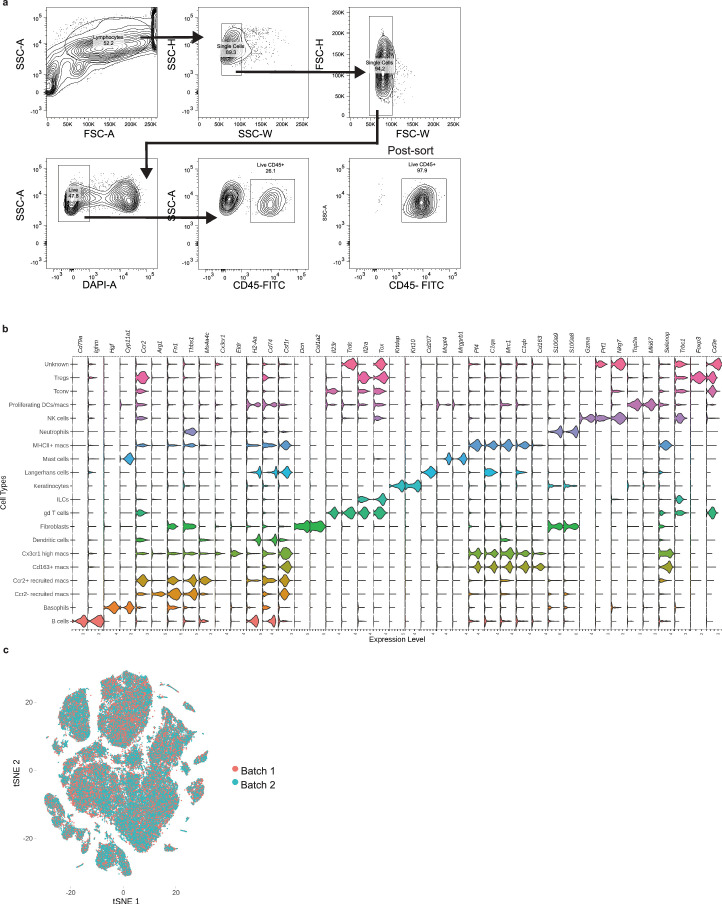
Single-cell RNA sequencing analysis of immune cells. (**a**) Gating strategy for CD45+ cells from digested skin single cell suspension. (**b**) Violin plot marker genes used to cell type annotation (**c**) t-SNE plot showing the overlap between the two biological replicates.

To determine if immune responses to injury underlay the kinetics of pain hypersensitivity, we isolated CD45^+^ immune cells 4, 24 and 48 h post-injury from mouse paw skin in all three models and healthy skin from the contralateral (CL) paw at *T*_max_ (4, 24 and 48 h for zymosan, incision and UV burn, respectively) and performed scRNA-seq to identify injury-induced gene programs (Extended Data Fig. 1a and Supplementary Table 1). To classify the cell types across all conditions, we integrated the data and performed a t-distributed stochastic neighbor embedding (t-SNE) dimensionality reduction (Fig. 1b). We used singleR for automated cell type detection (Supplementary Table 2). Because a full dataset of immune cells in inflamed skin has not been generated before, we complemented the singleR predictions with specific cluster annotations based on current knowledge of skin immune populations^7^ (Fig. 1b and Extended Data Fig. 1b). From the scRNA-seq data merged from all injuries and time points, we also identified three subsets of dermal macrophages annotated as *Cx3cr1*^hi^ Macs, *Cd163*^+^ Macs and *MHCII*^+^ Macs based on common expression of *Cd64*, *Selenop* and *Mrc1*, and unique expression of *Cx3cr1, Cd163* and *H2-Ab1*, respectively (Fig. 1b and Extended Data Fig. 1b), consistent with three transcriptionally distinct subsets of dermal macrophages in healthy skin^7^. We also identified dendritic cells (DCs) and Langerhans cells (*H2-Ab1*, *Cd74* and *Csf1r*), conventional T cells (T_conv_ cells, *Cd3e* and *Trbc1*), regulatory T cells (T_reg_ cells, *Cd3e* and *Foxp3*), gd T cells (*Trdc*), innate lymphoid cells (ILCs) (*Tox*, no *Cd3e*), B cells (*Cd79a* and *Ighm*), natural killer (NK) cells (*Prf1* and *Gzma*), neutrophils (*S100a9*), mast cells (*Mcpt4*) and basophils (*Hgf* and *Cyp11a1*) (Extended Data Fig. 1b). Two macrophage populations increased at *T*_max_ in the zymosan, incision and UV burn paws compared to zymosan_CL, incision_CL and UV burn_CL respectively, and based on their expression of *Ccr2*, we defined them as *Ccr2*^+^ and *Ccr2*^−^ recruited macrophages (recMacs) (Fig. 1c). A small keratinocyte and fibroblast cluster was also detected, due to a less than 100% purity of sorted CD45^+^ cells (Extended Data Fig. 1a). Cells from biological replicates showed minimal batch-to-batch variation (Extended Data Fig. 1c). As such, single-cell transcriptomics of normal and injured skin identified a broad spectrum of functionally diverse resident and infiltrating immune cells.

**Extended Data Fig. 2:**
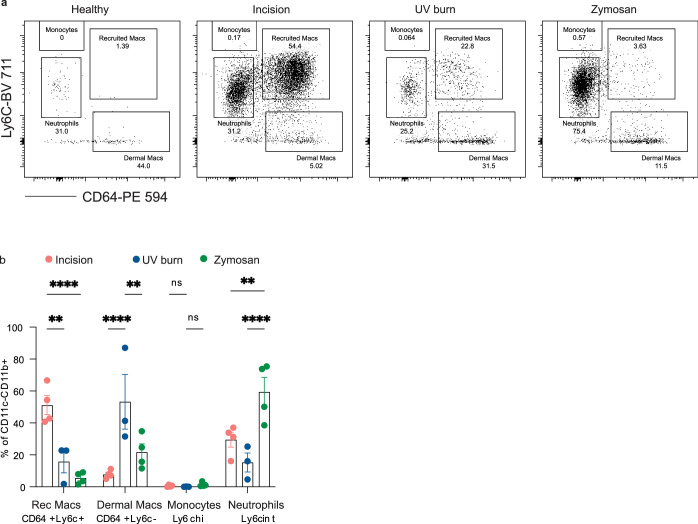
Different injuries show distinct myeloid cell populations at Tmax. Immune cells were isolated from injured or healthy paw skin and stained for various myeloid cell markers. (**a**) Immune cells isolated from injured paw skin stained for myeloid cell markers. Representative flow plots are gated on Live CD45 + CD11b + CD11c-cells. (**b**) Quantification of myeloid cell populations in incision (24 h) (n = 4), UV burn (48 h) (n = 3), and zymosan (4 h) (n = 4). Data is gated on live CD45 + CD11b + CD11c- cells. Data are represented as mean value ± SEM. p values calculated using one-way ANOVA, Tukey’s multiple comparison test. * p < 0.05, ** p, 0.01, *** p < 0.001,*** p < 0.0001, ****<0.00001 and n.s. = not significant.

We then used a scCODA proportionality analysis to determine how the immune population evolved post-injury in relation to pain hypersensitivity kinetics (Supplementary Tables 3–5). Proportions of neutrophils and *Ccr2*^+^ and *Ccr2*^−^ recMacs increased significantly 4 h post-zymosan and post-incision compared to CL controls (Fig. 1c,d), while these populations did not increase significantly in UV burn until 48 h compared to UV burn_CL (Fig. 1c,d). This suggested that infiltration of neutrophils and recMacs contributed to the development of heat hypersensitivity in each injury model. The change in the proportion of myeloid cells at *T*_max_, identified by the transcriptional profiling, mirrored that obtained by flow cytometry, which identified neutrophils as Ly6c^int^CD64^−^ and recMacs as Ly6c^+^CD64^+^ (Extended Data Fig. 2a,b). NK cells and basophils also increased in zymosan at *T*_max_ compared to zymosan_CL, which resolved at 24 h post-injury (Fig. 1c and Supplementary Table 3). NK cell proportions also increased at 4 h post-incision compared to incision_CL and were resolved by *T*_max,In_ (Fig. 1c and Supplementary Table 4).

Changes in skin-resident immune populations were observed and depended on the type of injury. *Cd163*^*+*^ Macs were significantly reduced in zymosan and incision at all time points compared to CL controls (Fig. 1c). Langerhans cells were significantly reduced at *T*_max,In_, compared to incision_CL (Fig. 1c and Supplementary Table 4). In UV burn, the proportion of ILCs decreased, while *Cx3cr1*^*hi*^ Macs, *MHCII*^*+*^ Macs and DCs increased at *T*_max,UV_ compared to UV burn_CL (Fig. 1c and Supplementary Table 5). As such, the infiltration of neutrophils and recMacs broadly correlated with pain development across injury types, while changes in the proportions of tissue-resident immune cells were injury specific.

### Macrophage transcriptional changes mirror pain hypersensitivity

**Fig. 2:**
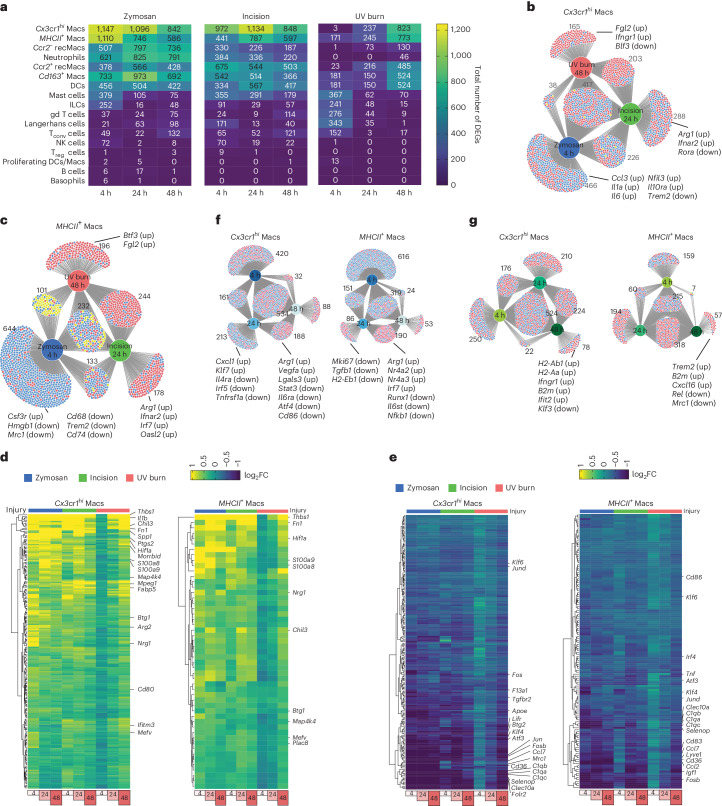
Macrophage transcriptional changes mirror pain hypersensitivity. **a**, Heatmaps showing the total number of differentially expressed genes (upregulated and downregulated combined) compared to CL in each immune cell type. For differentially expressed genes (DEGs), log_2_FC threshold of 0.25 and min.pct of 0.1 was applied. **b**,**c**, DiVenn plots showing the overlap of DEGs in different injuries at *T*_*max*_ (zymosan at 4 h, incision at 24 h and UV burn at 48 h) compared to CL in *Cx3cr1*^hi^ Macs (**b**) and *MHCII*^*+*^ Macs (**c**). **d**,**e**, Heatmaps showing the log_2_FC of the upregulated DEGs common in zymosan at 4 h, incision at 24 h and UV burn at 48 h compared to CL controls in *Cx3cr1*^hi^ Macs (**d**) and *MHCII*^*+*^ Macs (**e**). **f**, DiVenn plots showing the overlap of DEGs in zymosan at different time points of 4 h, 24 h and 48 h compared to CL in *Cx3cr1*^hi^ Macs and *MHCII*^*+*^ Macs. **g**, DiVenn plots showing the overlap of DEGs in incision at different time points of 4 h, 24 h and 48 h compared to CL in *Cx3cr1*^hi^ Macs and *MHCII*^*+*^ Macs. In **b**, **g** and **h**, the red circle denotes upregulated, the blue circles denote downregulated and the yellow circle denotes divergent regulated genes.

Next, we investigated injury-induced gene programs in each immune cell type in the three injury conditions. The greatest number of differentially expressed genes compared to CL were found in the dermal macrophage subsets *Cx3cr1*^hi^ Macs and *MHCII*^+^ Macs (Fig. 2a), and the magnitude of this injury-induced transcriptional response peaked at *T*_max_ (Fig. 2a), indicating that the kinetics of the injury-triggered transcriptional changes in dermal macrophages mirrored the temporal development of pain hypersensitivity in each injury model. Comparison of differentially expressed genes in *Cx3cr1*^hi^ Macs (Fig. 2b) or *MHCII*^+^ Macs (Fig. 2c) showed significant numbers of genes unique to each injury type, as well as genes differentially expressed in all three injuries at *T*_max_ compared to CL. For example, in *Cx3cr1*^hi^ Macs, *Fgl2* and *Ifngr1* were only upregulated at *T*_max,UV_ compared to UV burn_CL, *Arg1* and *Ifnar2* were only upregulated in incision compared to incision_CL at *T*_max,In_, and *Ccl3*, *Il1a*, *Il6*, *Nfil3* were only upregulated at *T*_max,Zy_ compared to zymosan_CL (Fig. 2b). Similarly, in *MHCII*^*+*^ Macs, *Btf3* and *Fgl2* were specifically induced at *T*_max,UV_, *Arg1*, *Ifnar2*, *Irf7* and *Oasl2* were uniquely upregulated at *T*_max,In_, while *Hmgb1*, *Mrc1*, *Cd68* and *Trem2* were downregulated at *T*_max,Zy_ compared to the respective CL controls (Fig. 2c). This analysis revealed insights into injury-specific macrophage signatures associated with pain hypersensitivity, with upregulation of inflammatory genes and suppression of phagocytic genes in zymosan and upregulation of interferon-responsive genes in incision.

**Extended Data Fig. 3:**
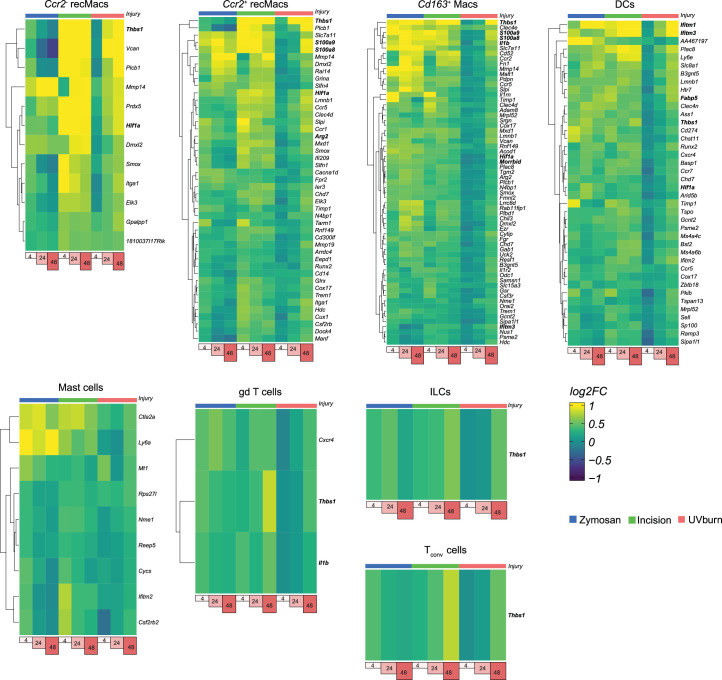
Immune cells in zymosan, incision and UV burn show commonly upregulated genes at *T*_*max*_. Heatmaps showing the log2 fold-change of the commonly upregulated differentially expressed genes common in zymosan, incision, and UV burn at Tmax compared to CL_zymosan, CL_incision and CL_UVB respectively in *Ccr2*^*-*^ recMacs, *Ccr2*^*+*^ recMacs, *Cd163*^*+*^ Macs, dendritic cells, mast cells, gd T cells, ILCs and T_conv_ cells.

We then analyzed the set of genes upregulated in *Cx3cr1*^hi^ Macs at *T*_max,UV_, *T*_max,In_ and *T*_max,Zy_ compared to their respective CLs (Fig. 2d). These included genes such as *Thbs1*, *Il1b*, *Chil3*, *Fn1*, *Spp1*, *Ptgs2*, *Btg1*, *Map4k4*, *Hif1a, Arg2* and *Ifitm3* (Fig. 2d). *Thbs1*, *Fn1*, *Hif1a*, *Btg1* and *Map4k4* were commonly upregulated in *MHCII*^*+*^ Macs at *T*_max,Zy_, *T*_max,In_ and *T*_max,UV_ compared to CL (Fig. 2d). At *T*_max,Zy_, *T*_max,In_ and *T*_max,UV_, *Ccr2*^−^ recMacs upregulated *Thbs1* and *Hif1a*, *Ccr2*^*+*^ recMacs upregulated *Thbs1*, *Hif1a* and *Arg2*, while *Cd163*^*+*^ Macs upregulated *Thbs1*, *Il1b*, *Hif1a* and *Morrbid* (Extended Data Fig. 3). *Thbs1* was commonly upregulated in DCs, gd T cells, T_conv_ cells and ILCs at *T*_max_ in all three injury models (Extended Data Fig. 3). *Thbs1* (encoding the secreted protein TSP-1) and *Hif1a* (encoding the transcription factor Hif1a) could therefore be genes strongly associated with pain hypersensitivity. DCs also showed upregulation of interferon genes such as *Ifitm1*, *Ifitm2* and *Ifitm3* at *T*_max,Zy_, *T*_max,In_ and *T*_max,UV_. *Ifitm2* was also upregulated in mast cells at *T*_max_ in all injuries (Extended Data Fig. 3).

**Extended Data Fig. 4:**
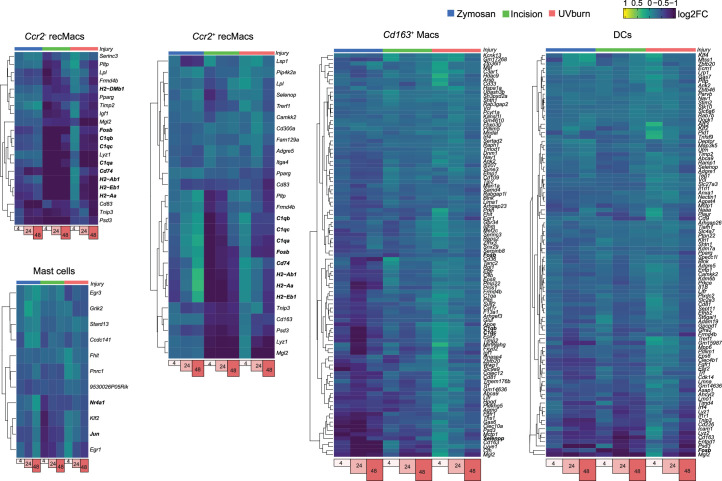
Immune cells in zymosan, incision and UV burn show commonly downregulated genes at *T*_*max*_. Heatmaps showing the log2 fold-change of the commonly downregulated differentially expressed genes common in zymosan, incision, and UV burn at Tmax compared to CL_zymosan, CL_incision and CL_UVB respectively in *Ccr2*^*-*^ recMacs, *Ccr2*^*+*^ recMacs, *Cd163*^*+*^ Macs, dendritic cells and mast cells.

Among the genes downregulated at *T*_max_ in all injuries, transcription factors encoding genes, including *Klf6*, *Atf3*, *Btg2*, *Klf4*, *Jun* and *Fos* were suppressed in *Cx3cr1*^*hi*^ Macs at *T*_max_ compared to CL (Fig. 2e). *Klf6*, *Irf4*, *Atf3* and *Fosb* were downregulated in *MHCII*^*+*^ Macs at *T*_max,Zy_, *T*_max,In_ and *T*_max,UV_ compared to CL (Fig. 2e). Several genes associated with anti-inflammatory macrophage program, including *C1qa*, *C1qb*, *C1qc*, *Selenop* and *Cd36*, were also suppressed in both *Cx3cr1*^*hi*^ and *MHCII*^+^ Macs at *T*_max,Zy_, *T*_max,In_ and *T*_max,UV_ compared to CL (Fig. 2e). *Fosb* was downregulated in *Ccr2*^−^ and *Ccr2*^*+*^ recMacs, *Cd163*^*+*^ Macs and DCs (Extended Data Fig. 4), while mast cells downregulated other CREB signaling targets, including *Nr4a1* and *Jun* (Extended Data Fig. 4) at *T*_max,Zy_, *T*_max,In_ and *T*_max,UV_. Similar to *Cx3cr1*^*hi*^ Macs and *MHCII*^+^ Macs, *Ccr2*^−^ and *Ccr2*^*+*^ recMacs downregulated *C1qa*, *C1qb* and *C1qc* at *T*_max_ in all injuries compared to CL (Extended Data Fig. 4). Genes associated with antigen presentation, including *H2-Ab1*, *H2-Eb1*, *H2-Aa* and *Cd74*, were also suppressed in Ccr2^−^ and Ccr2^+^ recMacs (Extended Data Fig. 4), suggesting suppression of genes regulated by CREB signaling or associated with the antigen presentation machinery associated with pain hypersensitivity.

Finally, we asked which gene programs were associated with pain resolution in zymosan and incision injuries by analyzing the transcriptional program unique to the pain-resolving time points (hereafter *T*_res_), which was 24 h post-injury in zymosan (*T*_res,Zy_) and 48 h post-injury in incision (*T*_res,In_) compared to CL controls. At *T*_res,Zy_, *Cx3cr1hi* Macs induced tissue repair genes, including *Arg1*, *Vegfa* and *Lglas3*, and suppressed *Il4ra, Il6ra, Stat3* and *Irf5* (Fig. 2f). Similarly, *MHCII*^*+*^ Macs showed upregulation of Arg1 and suppression of *Il6st*, *Nfkb1* and *Runx1* at *T*_res,Zy_ (Fig. 2f). *Trem2* and gene associated with antigen presentation (*H2-Ab1* and *B2m*) were upregulated at *T*_res,In_ in *MHCII*^*+*^ Macs and *Cx3cr1*^*hi*^ Macs, respectively (Fig. 2g), indicating that pain resolution was accompanied by suppression of pain-inducing gene programs and induction of pain-resolving gene signatures.

### DRG neurons show subtype-specific receptor expression profiles

**Fig. 3:**
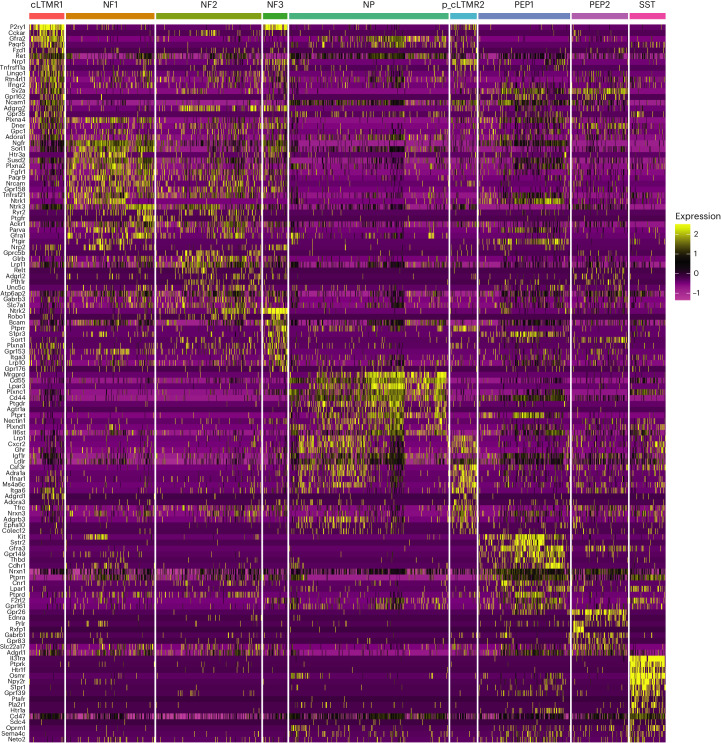
DRG neurons have subtype-specific receptor expression profiles. FindMarker analysis of receptor genes expressed by sensory neurons isolated from DRG in female mice based on snRNA-seq data, from ref. ^4^. Heatmaps show significantly (adjusted *P* < 0.05) differentially expressed receptor genes in neurons with log_2_FC of 0.5 and min.pct of 0.25.

**Extended Data Fig. 5:**
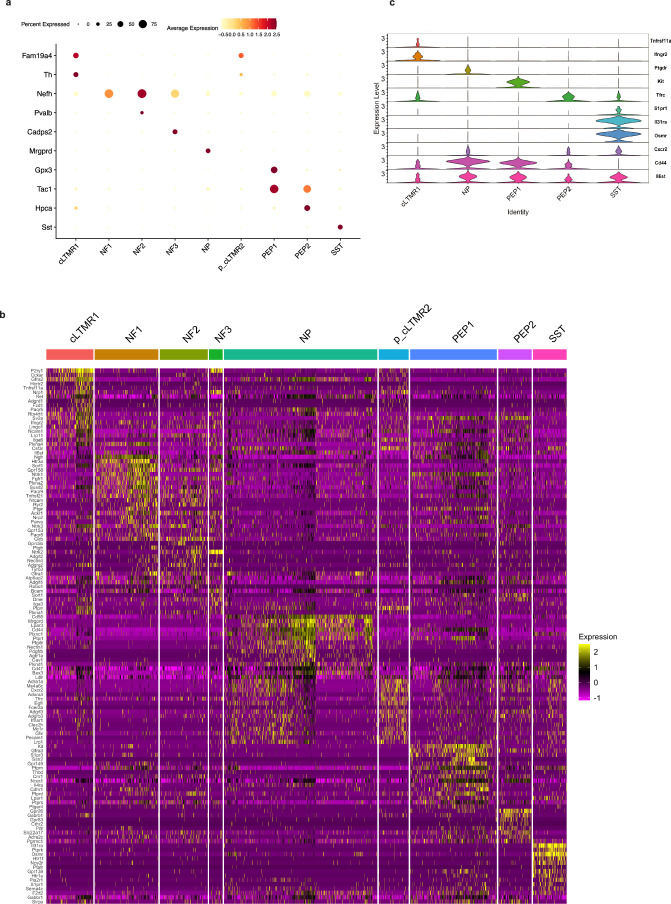
Receptor expression profile in male DRG neurons. (**a**) Dot plot showing marker genes used to annotate given DRG subtypes. (**b**) Differential expression of receptor genes in cLTMR1, NF1, NF2, NF3, NP, p_cLTMR2, PEP1, PEP2 and SST, and cLTMR cells from male DRG neurons, from ref. ^4^. Heatmaps show significantly (adjusted *p* < 0.05) differentially expressed genes in neurons with log_2_FC = 0.5 and min. pct. = 0.25. (**c**) Violin plot showing normalized expression of specific receptor genes in cLTMR1, PEP1, PEP2, NP and SST neurons.

To investigate whether DRG neurons expressed receptors that could enable interactions with immune cells, we utilized a published snRNA-seq transcriptomic dataset^4^ from DRG of 8–12-week-old healthy C57BL/6J wild-type male and female mice. DRG neurons do not exhibit transcriptional changes in response to acute inflammatory injury^4^; therefore, we complemented the healthy DRG transcriptome with immune cell transcriptomes in the zymosan, incision and UV burn inflammatory models at all time points. This consists of nine subsets of sensory neurons, including *Tac1*^*+*^*Gpx3*^*+*^ peptidergic type 1 neurons (PEP1), *Tac1*^*+*^*Gpx3*^*–*^ peptidergic type 2 neurons (PEP2), *Mrgprd*^*+*^ nonpeptidergic neurons (NP), *Sst*^*+*^ somatostatin neurons (SST), *Fam19a4*^*+*^*Th*^*+*^ c fiber low threshold mechanoreceptors (cLTMR1), Nefh^+^Pvalb^*–*^ neurons (NF1), Nefh^+^Pval^*+*^ neurons (NF2), Nefh^*+*^Cadps2^*+*^ neurons (NF3) and *Fam19a4*^*+*^Th^lo^ putative cLTMR (p_cLTMR2) (Extended Data Fig. 5a). These subsets are functionally distinct, with PEP, PEP2 and NP neurons activated in response to high-intensity mechanical or thermal stimuli, and SST neurons in response to itch. NF1, NF2 and NF3 are low-threshold mechanoreceptors^8^, while the precise function of cLTMR1 and p_CLTMR2 is not well defined (cLTMRs may be involved in mechanical pain)^9,10^. We performed a differential expression analysis in the DRG neurons of genes encoding proteins considered receptors to understand the ability of different DRG neurons to respond to external stimuli (Supplementary Data File 1). The receptor genes were obtained from the CellphoneDB database (also used for interactome analyses) (Supplementary Data File 1). This analysis showed that unique repertoires of receptors were expressed in different subsets of sensory neurons (Fig. 3). Subset-specific receptor expression profiles were observed in DRG neurons from both female (Fig. 3) and male (Extended Data Fig. 5b) mice. Many genes expressed in a subtype-specific manner encoded immune receptors (Fig. 3). For example, *Ifngr2* (encodes the receptor for type 1 cytokine interferon-γ (IFNγ)), was enriched in cLTMR neurons (Extended Data Fig. 5c), suggesting these neurons preferentially responded to IFNγ-producing cells, such as CD8^+^ T cells and NK cells, which are mediators of anti-viral and anti-tumor immunity. Expression of *Kit* (encodes KIT, a receptor for KITLG, which potentiates mast cell-mediated allergic inflammation)^11^, was observed only in PEP1 neurons (Extended Data Fig. 5c), suggesting these neurons amplify allergic immunity^12,13^ and that Kit signaling might sense allergic immune reactions. *Osmr*, which encodes the receptor for the oncostatin M ligand (OSM), was specifically expressed by SST neurons (Extended Data Fig. 5c), consistent with their role in OSM-mediated pruritus^14^. A small proportion of cLTMR1 neurons were enriched in the expression of *Tnfrsf11a*, which encodes RANK, a receptor for the TNF family member RANKL (encoded by *Tnfsf11*; Extended Data Fig. 5c). Expression of RANK in cLTMR1 neurons pointed to a potential role for neuronal intrinsic RANK signaling in pain^15^. Genes encoding several receptors traditionally studied in immune cells, such as *Cd44 (*encoding CD44) and *S1pr1* (encoding sphingosine-1-phosphate receptor 1), which mediate immune cell trafficking, were also expressed by DRG neurons (Extended Data Fig. 5c). While *Cd44* was broadly expressed by DRG neurons, *S1pr1* was highly expressed in SST neurons (Extended Data Fig. 5c), suggesting subset-specific receptor distribution. The differential expression of receptors for immune ligands by DRG neurons showed that different sensory neuron types can distinguish distinct immune cues.

### Macrophages are the strongest interactors of sensory neurons

**Fig. 4:**
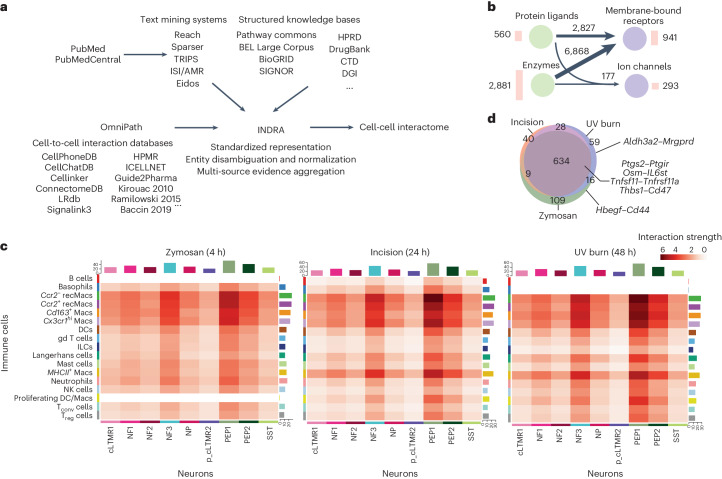
Macrophages are the strongest interactors of sensory neurons. **a**, An overview of the computational pipeline in which INDRA processes publications through multiple text mining systems and combines their output with structured knowledge bases integrated with INDRA directly, as well as the content of cell-to-cell interaction databases obtained via OmniPath to create an assembled cell–cell interactome. **b**, Summary of the modalities of the interactome derived from INDRA. Circles represent types of proteins: ligands, enzymes, membrane-bound receptors and ion channels. Numbers next to the circle represent protein types in the interactome. Arrows between circles show the number of distinct interactions among the corresponding protein type, with thicker arrows corresponding to a larger number of interactions. **c**, A heatmap of the interaction strength between immune cells (senders) and neurons (receivers) calculated for zymosan, incision and UV burn at *T*_max_. Color saturation represents the communication probability between the senders and receivers calculated by CellChat. **d**, Venn diagram depicting the number of shared and unique significant neuroimmune interactions for zymosan, incision and UV burn at *T*_max_.

**Extended Data Fig. 6:**
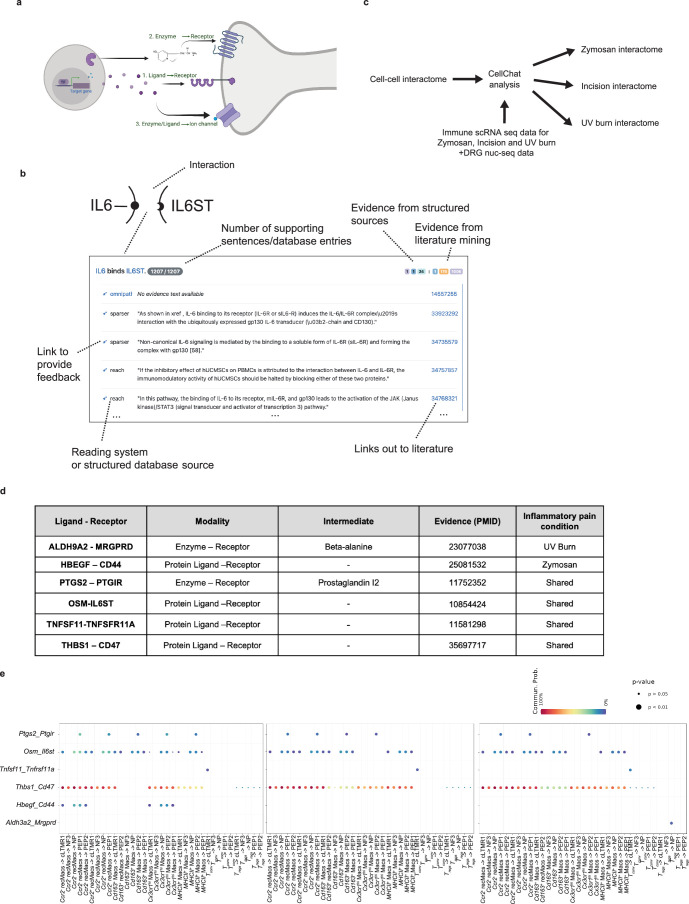
Neuroimmune interactomes of pain have injury-specific signatures. (**a**) Schematic showing the types of intercellular communication considered between immune cells and neurons for constructing the interactome. (**b**) Sample of the web interface for browsing evidence behind the cell-cell interactome. Each interaction is displayed as a heading summarizing the interaction (‘IL6 binds IL6ST’) with gene names linked to HGNC pages representing the gene. The total number of supporting pieces of evidence is shown, as is the breakdown of this number by specific source: different structured databases or literature mining systems integrated with INDRA. Each row under the heading represents a distinct database entry or sentence from a publication, with each publication linked to its corresponding PubMed landing page. Each row also links to a curation page where feedback can be given on the correctness of the interaction. (**c**) Overview of the condition-specific interactome construction. The general cell-cell interactome was provided as input to Cellchat together with data from immune cells for each of the three pain conditions: UVB, Zymosan, or incision, and combined with naïve DRG neuron data. This results in three condition-specific neuroimmune interactomes. (**d**) The modalities of interactions and the example of INDRA-based literature evidence, as shown by PMIDs for the representative interactions, are shown in Fig. 4d. (**e**) Dot plots showing examples of interactions between immune cells and DRG neurons that are shared or injury-specific.

To identify the full spectrum of possible interactions between immune cells and neurons, we used INDRA (integrated network and dynamical reasoning assembler) to assemble a systematic interaction knowledge base (interactome). INDRA builds a knowledge base from a variety of cell-to-cell interaction databases and through text mining of the scientific literature and combines duplicate and overlapping mentions of the same mechanism in a standardized format known as INDRA statements (Fig. 4a)^6,16^. INDRA standardized the names of genes and proteins to Human Genome Organization Gene Nomenclature (HUGO symbols), which was used for defining interactomes. We included three possible modalities of crosstalk between cell types in the interactome: ligands secreted or expressed on the surface of immune cells that physically interacted with cell surface receptors expressed on neurons (for example, the cytokine IL-6 interacting with its cognate receptor IL-6ST); metabolites whose production and secretion was controlled by enzymes in immune cells and could interact with cell surface receptors on neurons, creating indirect interactions between immune cell enzymes and neuronal receptors (such as the interaction between enzyme PTGS2, which controls production of prostaglandin E2, with the PTGER4 receptor); and proteins or metabolites secreted by immune cells that directly or indirectly interacted with ion channels expressed on the surface of neurons (for example, the interactome of the growth factor NGF with the ion channel TRPV1) (Extended Data Fig. 6a). We first identified a set of proteins considered to be ligands, receptors, enzymes or ion channels to include in the interactome, based on the following datasets: to identify the ligand and receptor proteins, we used the CellPhoneDB database^17^ (for each interaction, one interactor was classified as receptor and the other as ligand, based on a set of consensus direction information standardized from multiple sources by OmniPath, which aggregates multiple cell-to-cell interaction databases^18,19^ (Methods)); the enzyme list was constructed on the basis of the ExPASy Enzyme database^20^, which captures human proteins with enzymatic activity, after excluding kinases and phosphatases as intermediaries in signaling pathways not likely to be directly involved in intercellular interactions; finally, for ion channels, we used a list curated by the NIH Illuminating the Druggable Genome program (https://druggablegenome.net/). Together, these resources yielded 941 receptors (Supplementary Data File 1), 560 ligands (Supplementary Data File 2), 2,881 enzymes (Supplementary Data File 3) and 293 ion channels (Supplementary Data File 4).

We used these gene sets to assemble three potential interaction modalities. Ligand–receptor interactions were obtained from OmniPath, then aligned with text mining and structured knowledge evidence collected by INDRA to obtain 2,827 distinct interactions (Fig. 4c). We used the Pathway Commons database to identify products controlled by each enzyme and INDRA to find interactions between these products and receptors^21^, which created 6,868 total interactions between an enzyme’s product and a receptor (Fig. 4b and Supplementary Data File 5), while maintaining the underlying relationships between enzymes, products and receptors, which ensured that each interaction was traceable to the evidence from which it was derived (Extended Data Fig. 6b). Finally, we used INDRA to find direct or indirect effects on ion channels involving protein ligands or enzyme products, which added 177 interactions (Fig. 4b). In total, the final interactome contained 9,872 unique interactions (Fig. 4b and Supplementary Data File 6).

**Extended Data Fig. 7:**
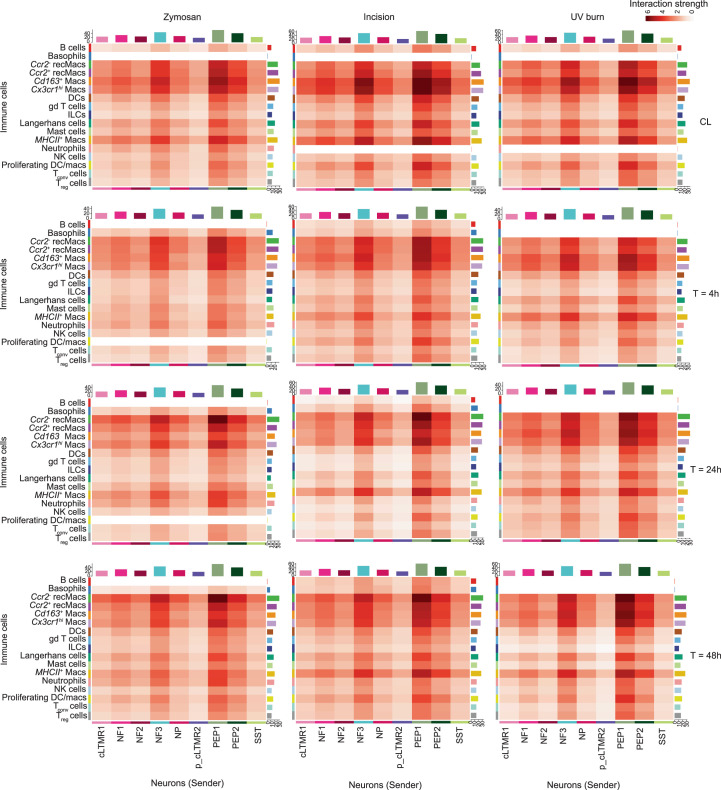
Immune cell – DRG neurons interactome show injury-specific signatures. Heatmap of the interaction strength between immune cells (senders) and neurons (receivers) calculated for zymosan, incision and UV burn at CL, 4 h, 24 and 48 h. Color saturation represents the communication probability between the senders and receivers calculated by CellChat.

Next, we mapped human genes in the INDRA interactome to mouse genes using ortholog mappings made available by HGNC, so the INDRA interactome can be applied to mouse skin immune scRNA-seq and DRG neuron snRNA-seq data. We complemented each immune scRNA-seq dataset for zymosan, incision and UV burn at 4, 24 and 48 h and CL conditions with snRNA-seq from steady-state DRG neurons^4^ and used CellChat to identify statistically significant interactions between each immune and neuronal cell type^22^ (Extended Data Fig. 6c). We focused primarily on interactions between immune cells as the source and neurons as receivers (Extended Data Fig. 7), as these are likely to modulate inflammatory pain. *Ccr2*^*+*^ and *Ccr2*^−^ recMacs and dermal macrophages (*Cx3cr1*^*hi*^ Macs, *MHCII*^*+*^ Macs and *Cd163*^*+*^ *Macs*) showed the highest interaction strength with DRG neurons in all three conditions at all time points (Extended Data Fig. 7). The DRG neurons PEP1, PEP2 and NF3 were the strongest interactors with immune cells across all immune cell types (Fig. 4c and Extended Data Fig. 7). The interactomes of incision_CL and UV burn_CL were similar to each other, with no interactions predicted between neutrophils and neurons. However, zymosan_CL showed interactions between neutrophils and DRG neurons (Extended Data Fig. 7), which could be due to zymosan leaking into the circulation and increasing neutrophils recruitment into healthy CL paw skin. We further analyzed a network of bidirectional interactions between immune and neuronal cell types at *T*_max,Zy_ (Supplementary Data File 7), *T*_max,In_ (Supplementary Data File 8) and *T*_max,UV_ (Supplementary Data File 9). The strength of the interaction between gd T cells or ILCs and neurons was reduced at *T*_max_ in all injuries compared to CL (Extended Data Fig. 7). The majority of neuroimmune interactions between immune cells (source) and neurons (receiver) were shared in all injuries at *T*_max_ (Fig. 4d and Extended Data Fig. 6d). This included *Ptgs2*–*Ptgir (Ptgs2* encodes prostaglandin synthase 2 (COX-2), the target of nonsteroidal anti-inflammatory drugs^22^), *Osm* (encoding the immune ligand oncostatin M)–*Il6st* (encoding the IL-6 receptor family protein, gp130)*, Tnfsf11–Tnfrsf11a* and *Thbs1* (encoding secreted thrombospondin-1, TSP-1)–*Cd47* (encoding the receptor CD47) (Fig. 4d). The cell types mediating these interactions, however, were dependent on the injury type. The cell pairs *Ccr2*^−^ recMacs–PEP1, *Ccr2*^*+*^ recMacs–PEP1, *Cd163*^*+*^ Macs–PEP2, *Cx3cr1*^*hi*^ *Macs–*PEP2 and *MHCII*^*+*^ Macs–PEP2 were predicted to mediate the *Ptgs2*–*Ptgir* interaction at *T*_max,Zy_ (Extended Data Fig. 6e), while the cell pairs *MHCII*^*+*^ Macs–PEP2 and the cell pairs *Cd163*^+^ Macs–PEP2 and *MHCII*^*+*^Macs–PEP2 did not show *Ptgs2*–*Ptgir* interactions at *T*_max,Zy_ or *T*_max,UV_, respectively (Extended Data Fig. 6e). A *Osm–Il6st* interaction was predicted to be mediated by *Ccr2*^−^ recMacs, *Ccr2*^*+*^ recMacs, *Cd163*^*+*^ Macs, *Cx3cr1*^*hi*^ Macs, *MHCII*^*+*^ Macs and cLMTR1, NP, PEP1 and PEP2 neurons at *T*_max,Zy_ (Extended Data Fig. 6e), but not by *Cx3cr1*^*hi*^ Macs at *T*_max,In_ and at *T*_max,UV_ (Extended Data Fig. 6e). A *Thbs1–Cd47* interaction was predicted between all macrophage types and cLMTR1, NF3, NP, PEP1 and PEP2 neurons at at *T*_max,In_ and at *T*_max,UV_ (Extended Data Fig. 6e). At *T*_max,Zy_, all macrophage subsets except *Cd163*^*+*^ Macs mediated *Thbs1–Cd47* interactions. *Tnfsf11–Tnfrsf11a* interaction was predicted to be mediated by T_conv_ cells and cLTMR1 neurons in all three injuries at *T*_max_ (Extended Data Fig. 6e).

The analysis also predicted interactions specific to each inflammatory pain model (Extended Data Fig. 6d). Interactions between *Hbegf* (encoding an inducer of mechanical hypersensitivity) in *Ccr2*^−^ recMacs and *Cx3cr1*^*hi*^ Macs, and *Cd44* in cLTMR1, NP, PEP1 and PEP2 neurons was uniquely found at *T*_max,Zy_ (Extended Data Fig. 6e). Interactions between *Aldh3a2*, which encodes aldehyde dehydrogenase 3, in T_reg_ cells and *Mrgprd* occurred at *T*_max,UV_ (Fig. 4d and Extended Data Fig. 6d,e). *Aldh3a2* controls the production of beta-alanine, an itch inducer^23^, and this interaction might underlie UV burn-specific mechanisms of itch (Extended Data Fig. 6e). Thus, interactome analysis predicted known pain and itch pathways such as *Ptgs2*–*Ptgir, Osm–Il6st, Tnfsf11–Tnfrsf11a* and *Aldh3a2–Mrgprd*, and previously unappreciated neuroimmune interactions in the periphery, including *Thbs1–Cd47*.

### TSP-1 inhibits PGE2-mediated nociceptor sensitization

**Fig. 5:**
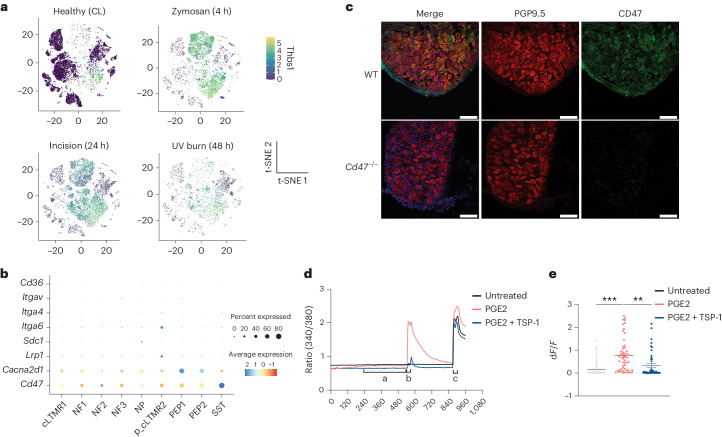
TSP-1 inhibits PGE2-mediated nociceptor sensitization. **a**, t-SNE plot of normalized *Thbs1* expression in immune cells from healthy (CL) and inflamed skin following zymosan, incision and UV burn injury at *T*_max_. **b**, Dot plot showing the expression of TSP-1 receptors (*Cd47*, *Cacna2d1*, *Lrp1*, *Sdc1*, *Itga6*, *Itga4*, *Itgav* and *Cd36*) in DRG neuron subtypes (cLTMR1, NF1, NF2, NF3, NP, p_cLTMR2, PEP1 and PEP2) in wild-type (WT) healthy mouse lumbar DRG^4^. **c**, The expression of CD47 on frozen wild-type or *Cd47*^−/−^ DRG neuron sections stained with PGP9.5. Scale bar, 100 μm. Representative of two independent experiments. **d**, Fura-2-based calcium imaging in cultured sensory neurons from DRG obtained from WT male mice treated with SES, 1 µM PGE2 or 1 µM PGE2 + 200 ng ml^−1^ TSP-1 for 7 min, followed immediately by 100 nM capsaicin, 100 nM capsaicin + 1 µM PGE2 or 100 nM capsaicin + 1 µM PGE2 + 200 ng ml^−1^ TSP-1, respectively for 30 s, a 5 min SES wash and treatment with 1 µM capsaicin for 30 s. SES was used as the recording solution. Treatments were applied during live imaging using a gravity-based perfusion system. Frames were captured every 3 s. Intensity traces of ratio of 340/380 are plotted. **e**, d*F*/*F* calculated as (*F*_1_ − *F*_0_)/*F*_0,_ where *F*_1_ is the peak response within 40 s of treatment with 100 nM capsaicin and *F*_0_ is the average of 10 s before the PGE2-treatment time point. SES, *n* = 80; PGE2, *n* = 55; PGE2 + TSP, *n* = 42. All TRPV1^+^ neurons are from one experiment consisting of three separate recordings for each condition. Data are represented as mean value ± s.e.m. Unpaired two-tailed *t*-test was performed. *P* < 0.05 was considered significant. An individual dot represents a neuron that was included in the analysis. Data are representative of three independent experiments.

**Extended Data Fig. 8:**
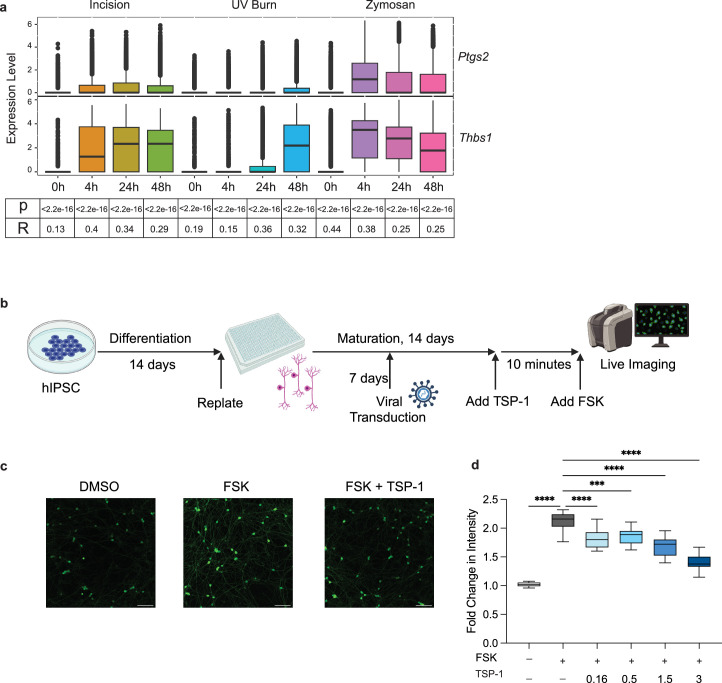
Human TSP-1 inhibits PKA activity in human sensory neurons. (**a**) Box plot of Ptgs2 and Thbs1 expression showing normalized expression of the two genes. Upregulation of Thbs1 mirrors pain hypersensitivity as observed in Fig. 1a. The p-values and correlation coefficients indicated below are calculated using the Pearson correlation analysis for the average expression *Thbs1* and *Ptgs2* at each timepoint per condition. (**b**) Timeline of iPSC-derived sensory neuron differentiation, viral transduction and experimental treatments. (**c**) Representative images of iPSC-derived sensory neurons expressing PKA reporter (green) treated with DMSO, forskolin (FSK), or FSK + TSP1. The scale bar is 100 μm. Representative of two independent experiments with at least 9 wells in each condition. (**d**) Box plot graph showing the fold change (post/pre-treatment) in fluorescent intensity of PKA reporter in cells treated with FSK alone or FSK in the presence of escalating doses of TSP1. DMSO, n = 9; FSK, n = 18; TSP1 0.16 ug/ml, n = 9; TSP1 0.5 ug/ml, n = 9; TSP1 1.5 ug/ml, n = 9; TSP1 3 ug/ml, n = 9; TSP1 5 ug/ml, n = 9; TSP-1 10 ug/ml, n = 9 Bounds of box and whiskers are 25% and 75% percentile with 95% confidence level. One-way ANOVA was performed (n = 3 independent experiments), **p < 0.005, ***p < 0.001, ****p < 0.0001.

TSP-1 interacts with CD47 on platelets to trigger Gi-coupled GPCR signaling, which attenuates the activation of the PKA kinase^24,25^. Expression of *Thbs1* increased in neutrophils, *Ccr2*^*+*^ recMacs, *Ccr2*^−^ recMacs, *MHCII*^*+*^ Macs, *Cx3cr1*^*hi*^ Macs, DCs, ILCs and T_conv_ cells at *T*_max,Zy_, *T*_max,In_ and *T*_max,UV_ (Fig. 5a). Expression of *Thbs1* correlated with the expression of *Ptgs2* across all cells in all three injuries at all time points and mirrored the development of pain hypersensitivity in all injuries (Extended Data Fig. 8a), suggesting that neurons were concurrently exposed to both TSP-1 and pain-sensitizing molecules like prostaglandin E2. Expression of *Cd47* in mouse DRG neurons was higher than other TSP-1 receptors^26^ (Fig. 5b) and was enriched in DRG neurons that drive pain or itch (PEP1, PEP2, NP and SST) compared to low-threshold mechanoreceptors (NF1, NF2 and NF3) (Fig. 5b), indicating a potential role for CD47 signaling in regulating nociception. We also confirmed that CD47 was expressed on mouse DRG neurons (Fig. 5c). In nociceptors, activation of PKA triggers several sensitization pathways by direct phosphorylation of TRPV1 and Nav1.8 channels, which lowers channel activation thresholds^27–29^. To test whether TSP-1 signaled through CD47 to modify PKA-mediated TRPV1 sensitization in DRG neurons, we used a capsaicin-induced sensitization assay in cultured mouse DRG neurons. Capsaicin-responsive, TRPV1^+^ nociceptor neurons were exposed to PGE2 as a sensitizing agent, and intracellular calcium levels were measured using the calcium-sensitive fluorescent dye, Fura-2. The amplitude of Ca^2+^ influx triggered by low-dose (100 nM) capsaicin was significantly higher in DRG neurons treated with PGE2 for 7 min than in untreated neurons (the majority of DRG neurons did not respond to low-dose capsaicin without prior sensitization; Fig. 5d,e), indicating that PGE2 treatment reduced TRPV1 activation thresholds. DRG neurons co-treated with PGE2 and TSP-1 for 7 min had a significant attenuation of the TRPV1 sensitization, compared to PGE2-only treated neurons, as indicated by reduced Ca^2+^ influx in response to low-dose capsaicin (Fig. 5d,e). To test whether TSP-1 also suppressed sensitization of human sensory neurons, we generated human induced pluripotent stem (iPS) cell-derived sensory neurons^30^ and used lentiviral transfection to express a fluorescent-based PKA activity sensor under a human Synapsin promoter to assess neuronal-specific PKA activity as indicated by GFP channel fluorescent intensity (Extended Data Fig. 8b). A significant induction of PKA activity immediately followed treatment with forskolin (FSK), an established PKA activator (Extended Data Fig. 8c,d). Pretreatment of iPS cell-derived sensory neuron culture with human TSP-1 for 10 min before FSK treatment decreased fluorescence of the PKA sensor in a dose-dependent manner (Extended Data Fig. 8c,d), suggesting a TSP-1-specific activity on neurons (Extended Data Fig. 8d). These findings validated predictions from the interactome model and highlighted a noncanonical role for TSP-1 in counteracting nociceptor sensitization.

## Discussion

Here, we generated a comprehensive dataset of immune changes in the skin at the single-cell level as inflammatory pain hypersensitivity developed and resolved, which can serve as a resource to decipher cell subsets and immune mediators that govern specific types of acute inflammatory pain. In addition to revealing gene programs associated with pain development, we also found that pain resolution is not simply the absence of pain-inducing gene programs but includes the induction of novel immune gene signatures suggesting an active process. We highlighted representative genes significantly differentially expressed in individual immune cell types at *T*_max_ and *T*_res_ post-injury. The online portal http://painseq.shinyapps.io/immune/ will enable further interrogation of immune gene programs responsive to various skin injuries.

The INDRA neuro-immune dataset was designed to be a general resource that contains as many potential interactions between immune cells and neurons as possible. These interactions were captured in a standardized intermediate format (INDRA statements), maintaining the provenance of the information^6^, which can be web-accessed. Several immune cell–nociceptor interactions predicted by the neuroimmune interactome at *T*_max_, such as *Ptgs2–Ptgir*, *Aldh3a2–Mrgprd* and *Hbegf–Cd44* (refs. ^2,23,31^) are known to promote pain or itch by enhancing nociceptor activity, underscoring the predictive power of INDRA-based platform. The immune ligand TSP-1, in contrast, suppressed PGE2-induced nociceptor sensitization. In the central nervous system, TSP-1 is produced by astrocytes and promotes synaptogenesis^32^. TSP-1 also acts on endothelial cells to suppress vascularization and nitric oxide production and promotes platelet aggregration^24,26^. We now report a role for TSP-1 in the peripheral nervous system. While the endogenous opioid system inhibits pain via actions on peripheral and central neurons that express the mu-opioid receptor (MOR) largely by reducing synaptic transmission^33^, TSP-1 reduces the sensitization of the peripheral terminals of sensory neurons. Because neutrophils, *Ccr2*^*+*^ and *Ccr2*^−^ recMacs that produced TSP-1 also secrete pro-nociceptive inflammatory mediators such as PGE2, IL-1β and TNF, TSP-1 is likely to fine-tune pain thresholds by the combined presence of both enhancers and suppressors of nociceptor sensitization in the complex immune milieu that develops in skin inflammation. This unbiased receptor–ligand interactome-based approach provided, therefore, a means for the comprehensive interrogation of exactly how the immune microenvironment encodes injury-specific and temporally regulated cues that are received by nociceptors to either heighten or reduce inflammatory pain sensitivity.

## Methods

### Mice

Eight-to-12-week-old C57BL/6J mice were obtained from the Jackson Laboratory (JAX:000664) as were Cd47-deficient mice (Jax:003173)^34^. Both male and female mice were used in all the behavior experiments. scRNA-seq was performed on female mice. No statistical methods were used to predetermine sample sizes, but our sample sizes are similar to those reported in previous publications^35,36^. All animal experiments were conducted according to institutional animal care and safety guidelines at Boston Children’s Hospital and Harvard Medical School.

### Inflammatory pain models

For each model, mice were randomly selected from the cage to receive the inflammatory stimulus. All mice in a cage received the same inflammatory stimulus, and the person inducing the inflammation was different from the person testing for hypersensitivity who was fully blinded.

#### Paw skin Incision

The mice were anesthetized by administration of 2.5% isoflurane. A 3–5 mm skin incision was made using a surgical sterile scalpel on the plantar surface of the mouse paw without cutting through the underneath muscle. The skin was sutured using 6–0 silk surgical suture (Ethicon, K889H) in an aseptic manner. The CL control was left untouched.

#### UV burn

Mice were anesthetized by administration of 2.5% isoflurane. Mouse hind paws were exposed to UV irradiation at an intensity of 1 J cm^−2^ for 2 min using a wavelength 305–315 nm fluorescent UV-B light source. The CL control was not exposed to UV.

#### Zymosan

The mice were anesthetized by administration of 2.5% isoflurane. Twenty microliters of 5 mg ml^−1^ zymosan (in saline) was injected into the plantar surface of the hind paw. Saline was injected into the CL control.

### Hargreaves thermal testing

The mice were habituated to the Hargreaves’s apparatus (IITC #390G) consisting of a glass floor heated to 30 °C and a plexiglass chamber for 2 days before testing, 1 h per day. On the day of assessment, the mice were habituated for another 1 h before the test. A focused radiant heat light (15% intensity) source was focused on the plantar surface of the left paw of mice, and a ramping heat stimulus was applied until a paw withdrawal was recorded. Readings were averaged from two trials. Blinded testing of mice with paw incision and zymosan injection was not possible since it was clear to see which paw was inflamed, but the operator was not told which foot the UV burn had been applied to, thus aiding blinding for those mice.

### Tissue processing of mouse plantar skin

The planter skin of the mouse hind paw was dissected, separating the muscle and collected into 1% bovine serum albumin (BSA) containing RPMI. The skin was minced using scissors into 1–2 mm pieces. Liberase TM (Roche) was added to the medium at a final concentration of 0.5 mg ml^−1^. Tissue was digested at 37 °C while vortexing at 400*g* for 90 min. The digested tissue was strained using a 100 µM strainer to obtain a single-cell suspension used for flow sorting on BD AriaII and single-cell transcriptomics.

### Flow cytometry

Cells were washed with FACS buffer and incubated with Fc block for 10 min on ice. Cells were then stained with mouse antibodies against mouse flow cytometry antibodies include CD45-FITC (1:400, BioLegend, cat. no. 103107), CD64-PE-594 (1:600, BioLegend, cat. no. 139319), CD11C-APC (1:400, BioLegend, cat. no. 101211) Cd11b-eFluor 780 (1:400, eBioscience, cat. no. 47-0112-82), Ly6G-PE (1:800, BioLegend, cat. no. 127607) and Ly6c-BV711 (1:2,000, BioLegend, cat. no. 128037) for 30 min on ice. Cells were washed and then resuspended with 3 µM 4′,6-diamidino-2-phenylindole (DAPI) for analysis. All flow cytometry analysis was performed on BD Fortessa. All data analysis was performed on FlowJo 10.

### scRNA-seq of immune cells (10x Genomics)

For isolation of immune cells for single-cell sequencing, cells were washed with FACS buffer and incubated with Fc block for 10 min on ice. Cells were then stained with anti-mouse CD45-FITC antibody for 30 min at 4 °C. CD45^+^ DAPI-negative cells were sorted to a 90% purity using BD ARIAII at Boston Children’s Hospital Flow cytometry core. Cells were collected into 0.5% BSA containing phosphate-buffered saline (PBS) without EDTA. Single-cell suspensions were encapsulated into droplets using the Chromium Next GEM Single Cell 3′ Reagent kit v3.1 (Dual Index).

### scRNA-seq library preparation

scRNA-seq libraries were prepared using Chromium Next GEM Single Cell 3′ Reagent Kit v3.1 (10x Genomics), following the manufacturer’s protocols. Briefly, to generate single-cell gel-bead-in-emulsion (GEM) solution, sorted cells were resuspended in a final volume of 40 μl and were loaded on a Next GEM Chip G (10x Genomics) and processed with the 10x Genomics Chromium Controller. Reverse transcription was performed as instructed: 53 °C for 45 min and 85 °C for 5 min in a thermocycler. Next, first-strand complementary DNA was cleaned with DynaBeads MyOne SILANE (10x Genomics, 2000048) and then amplified, followed by cleanup with SPRIselect Regent kit (Beckman Coulter, B23318). The cDNAs were examined on High-Sensitivity DNA Chip with Bioanalyzer (Agilent). Ten microliters of cDNA was forwarded to the library preparation. The dual-indexed libraries were examined on High-Sensitivity DNA Tape with TapeStation (Agilent) before being pooled for sequencing. The sequencing was performed with a NovaSeq 6000 (Illumina) at an estimated depth of 65,000–233,000 reads per cell. The raw scRNA-seq data were preprocessed using CellRanger v7.1.0 (10x Genomics), including aligning reads to the mouse reference genome (mm10-2020-A) and generating expression count matrices. The count matrices were further processed as described below.

### Initial quality control, clustering and visualization of scRNA-seq

Using the generated expression count matrices, an in-house scRNA-seq pipeline was built on the basis of the Seurat R package (R v4.2.3, Seurat v4.3.0)^37,38^, including quality control, cell filtering, spectral clustering, cell type annotation, differential gene expression and visualization. Multiplets were identified using the scds package (v1.14.0) using the cxds function^39^. Only identified singlets were kept for further analysis. We filtered out cells exhibiting extremely low or high library sizes and number of gene features, falling outside the 95% confidence interval, as well as those displaying high mitochondrial content (above the 5%). The cell counts pre- and post-filtering in each sample are included in Supplementary Table 1. Cells of good quality from the two replicates per group were merged, and the 12 experimental groups were integrated for analysis using Harmony^40^. Principal component analysis over the identified 2,000 highly variable genes was applied for data dimension reduction (dimensions 60) before cell clustering. Cell clustering was performed on integrated data with a shared nearest-neighbor graph-based method using the FindNeighbors function included in Seurat, followed by the Louvain algorithm for modularity optimization (resolution 2) using FindClusters function. After the cell clusters were determined, their top marker genes were identified with the FindAllMarkers function. For cluster annotation, the top marker genes based on the adjusted *P* value were manually curated to match canonical cell types and their marker genes based on literature research and public resources from scRNA-seq databases. The curated annotations are further supported by the automatic annotations using the SingleR tool (v2.0.0)^41^. For differentially expressed genes, the default parameters of the FindMarkers function from Seurat were used which is the Wilcoxon rank-sum test with log_2_fold change (FC) threshold of 0.25 and min.pct of 0.1 with adjusted *P* value <0.05.

### scCODA analysis

The cell-type compositional data analysis across the different injuries and time points using the scRNA-seq was carried out by using scCODA (v0.1.9)^42^. It uses a Bayesian approach along with a spike and slab before determining the credible effects on the basis of the inclusion probability. We used a false discovery rate cutoff of 0.05 to determine the significant proportion differences between the different groups on the basis of the credible effects.

### DiVenn plots

DiVenn tool (https://divenn.tch.harvard.edu/v2/) was used to compare the differentially expressed genes between different time points for selected cell types^43^. It visualizes the unique and common genes between time point comparisons in the form of networks. The upregulated and downregulated genes are marked as red and blue, respectively. The genes that are upregulated in one comparison and downregulated in others (or vice versa) are marked as yellow.

### Analysis of DRG neuron snRNA-seq data

DRG neuron snRNA-seq data were obtained from previously published data^4^. Neuronal cells from naive mice (male and female) were obtained by subsetting the Seurat object based on annotation. The neuron data are merged with the immune scRNA-seq data and are then scaled and normalized to be further used for the cell–cell communication analysis. For differential gene expression, the FindAllMarker function employing test.use = ‘wilcox’, Wilicoxon rank-sum test was used. A threshold of log_2_FC 0.8 and min.pt 0.25 and adjusted *P* < 0.05 was applied.

### Assembling the cell–cell interactome

INDRA 1.21.0 was used to assemble the interactome. The list of receptors was obtained from CellPhoneDB 2.1.7, using the protein_generated.csv file generated by CellPhoneDB’s generate_proteins function. OmniPath interactions were obtained on 22 March 2022, through INDRA’s OmniPath API, which uses the http://omnipathdb.org/interactions endpoint of the OmniPath web service to obtain the ‘ligrecextra’ subset of interactions corresponding to ligand–receptor interactions. These interactions were then filtered for curation effort >0, to ones containing human proteins only, and to the following OmniPath source identifiers corresponding to sources of cell–cell interaction information: ‘CellPhoneDB’, ‘Guide2Pharma’, ‘HPMR’, ‘ICELLNET’, ‘Kirouac2010’, ‘CellTalkDB’, ‘CellChatDB’, ‘connectomeDB2020’, ‘Ramilowski2015’ and ‘talklr’. The ligand list was derived by taking the ‘source’ participant of OmniPath interactions with a well-defined consensus direction and excluding known receptors. The ion channel list was derived from a curated list of proteins from the Illuminating the Druggable Genome project at https://druggablegenome.net/, removing overlaps with any receptors and ligands. The enzyme list was obtained from Expasy at ftp://ftp.expasy.org/databases/enzyme/enzyme.dat via PyOBO (https://github.com/pyobo/pyobo) to extract proteins that belong to any enzyme class, then filtered out any proteins known to be kinases or phosphatases on the basis of lists maintained by INDRA. Ligand–receptor interactions were taken from the overall OmniPath interaction list by filtering to interactions containing one ligand and one receptor. Evidence from publications and further structured databases aligned with these interactions was then obtained via INDRA. Ligand–ion channel interactions were obtained from INDRA directly by filtering Complex and Activation INDRA Statement types and Statements containing one ligand and one ion channel per the gene lists above. Statements were also filtered to ones supported by structured databases or at least two supporting sentences from text mining. Interactions involving enzymes were obtained in two parts. First, Pathway Commons v12 data were obtained in SIF format from https://www.pathwaycommons.org/archives/PC2/v12/PathwayCommons12.Detailed.hgnc.sif.gz and filtered to controls-production-of interaction whose controller is in the list of enzymes. The set of products for each enzyme was determined from the collection of rows remaining after these filters. For each enzyme product, INDRA was then used to find Activation and Complex Statements in which the enzyme product interacts with a receptor or an ion channel. Finally, enzyme–receptor and enzyme–ion channel interactions were generated by connecting an enzyme to a receptor or ion channel if its product interacts with the given receptor or ion channel. Finally, the interactome was exported into a tabular format compatible with CellChat using UniProt IDs to identify interacting proteins.

### Identification of significant interactions for cell–cell pairs

We used CellChat (v1.6.1) along with the assembled database of ligands and receptor pairs for mice to identify patterns of cell-to-cell communication^22^. To assess the likelihood of communication, we followed the methodologies outlined in the original research paper by Jin et al.^22^. We applied these methods at the ligand–receptor pair level. To ensure reliable and significant findings, communication between cell types observed in fewer than ten cells with adjusted *P* values greater than 0.05 were excluded. The plots were generated using the built-in functions within CellChat.

### Isolation and in vitro culture of DRG neurons

Lumbar and thoracic DRG were obtained from mice and collected in Dulbecco’s modified Eagle medium containing fetal calf serum and penicillin and streptomycin (DMEM). DRG were digested in Collagenase A (Roche, 5 mg ml^−1^) and Dispase II (Roche, 1 mg ml^−1^) for 70 min. Digested DRG were triturated using large-, medium- and small-sized polished glass pipettes in DMEM containing DNase. The cells were resuspended in DMEM and overlaid on 10% BSA solution. The bilayer was centrifuged for 12 min at 1,000*g* at reduced acceleration and deceleration. The top two layers were discarded, and the cell pellet was collected. For Ca imaging, cells were cultured at the center of a PDL (500 µg ml^−1^) and Laminin (5 mg ml^−1^) coated 35 mm dish in Neurobasal A Media (Life Technologies, 10888-022) containing 2% B-27, penicillin, streptomycin, 10 μM arabinocytidine (Sigma) and GDNF (5 ng ml^−1^, Sigma-Aldrich, SRP3200). A total of 5,000–8,000 cells were plated in a 50–80 µl drop and replenished with 2 ml of medium per dish and incubated at 37 °C and 5% CO_2_ for 48 h before calcium imaging experiments.

### Calcium imaging of DRG neurons

After 48 h in culture, DRG neurons were loaded with 4 µg ml^−1^ of Fura2-AM (Invitrogen) by incubating for 50 min at room temperature (RT). Cells were then washed with standard extracellular solution (SES) twice and left in 2 ml of SES to be used as recording solution. Live imaging was performed on Nikon Eclipse Ti microscope (Nikon) with standard 340- and 380-nm filters controlled by a Ludl Mac6000 shutter using Nikon Elements software. Frames were recorded every 3 s. All imaging was performed at RT. Cell treatments were performed using a gravity-based perfusion system. Cells were simply treated with SES for 2 min to get stable images and remove perfusion related artifacts. Then, cells were exposed to with freshly prepared PGE2 (1 μM) ± TSP-1 (200 ng ml^−1^) for 7 min, followed by the application of a low concentration of capsaicin (0.1 μM) for 30 s with or without PGE2 or TSP-1. After a 5 min perfusion of SES, a high concentration of capsaicin (1 μM) was applied at RT for 30 s, to identify all capsaicin-sensitive TRPV1^+^ neurons.

### Immunohistochemistry

DRG were collected in PBS and then fixed in 4% paraformaldehyde on a shaker at 4 °C for 30 min. After fixation, DRG were moved to 30% sucrose overnight, then mounted in OCT and frozen. DRG were sectioned at 20 μm, mounted onto SuperFrost Plus microscope slides and stored at −20 °C. Slides were thawed for 30 min, then washed with PBS for 15 min. Slides were incubated in blocking buffer for 2 h at RT (10% donkey serum, 0.4% Triton-X, 0.05% Tween 20 and 1% BSA) and then incubated in primary antibody overnight at 4 °C (Rb anti-PGP9.5 abcam ab108986 1:500, goat anti-CD47 RnD Systems AF1866 1:200). Slides are then washed 3× in PBS and incubated in secondary antibody at RT for 2 h (donkey anti-Rb Cy3 Jackson ImmunoResearch 711-165-152 1:500, donkey anti-goat 647 Thermo Fisher A-21447 1:500). Slides were finally washed 3× with PBS and mounted with Prolong anti-fade DAPI medium. Stained slides were imaged on a Leica SP8 confocal microscope with a 63× oil objective. *Z*-stacks spanning the tissue were taken, and four adjacent fields were tiled. ImageJ was used to obtain the maximum intensity projection.

### Molecular cloning

The ExRai-AKAR2 PKA reporter was cloned into a lentiviral backbone under the human synapsin promoter from a previously reported construct^44,45^. The lentiviral vector used to overexpress the PKA sensor in our study, pLV[Exp]-SYN1>PKAsub/ExRai2:WPRE, was constructed and packaged by Vector Builder. The vector ID is VB220620-1230gnm, which can be used to retrieve detailed information about the vector on vectorbuilder.com.

### Human iPS cell-derived sensory neuron differentiation and viral transfection

This study was approved by the institutional review board at Boston Children’s Hospital (IRB-P00006313). Human iPS cells (Lonza, LiPS-GR1.1) were thawed and expanded in E8 medium (Thermo Fisher Scientific, cat. no. A1517001) for three passages before onset of differentiation. iPS cells were passaged using the ReLeSR agent (Stem Cell Technologies, cat. no. 05872) and coated on vitronectin substrate (Thermo Fisher Scientific, A14700). iPS cells were differentiated into sensory neurons following previously established protocols^30^. In brief, iPS cells were plated in six-well plates at a density of 1.5 M cells per well and treated with 0.2 µM CHIR-98014 (SelleckChem, cat. no. S2745) and 2 µM A83-01 (Tocris, cat. no. 2939/10) in E6 medium (Thermo Fisher Scientific, cat. no. 1516401) for 3 days. Cells were then replated at 5.5 M cells per well in six-well AggreWell plates (Stem Cell Technologies) and further differentiated for 11 days in E6 medium containing 0.5 µM CHIR-98014, 2 µM A83-01, 1 µM DBZ (Tocris, cat. no. 4489/10) and 25 nM PD173074 (Tocris, cat. no. 3044/10). On day 14 from the onset of differentiation, nocispheres were dissociation using the MACS EB dissociation kit (Miltenyi Biotech, cat. no. 130-096-348) into single cells. Cells are then frozen in Neuron Freezing Media (Cell Applications, 042-50) or plated at 5,000 neurons per well in 384-well plates in neuron maturation medium containing DMEM/F12 (Thermo Fisher Scientific, cat. no. 11320082), B27 Supplement (Thermo Fisher Scientific, cat. no. a3353501), N2 Supplement (Thermo Fisher Scientific, cat. no. A1370701) with 25 ng ml^−1^ BDNF (Thermo Fisher Scientific, cat. no. PHC7074), 25 ng ml^−1^ GDNF (Thermo Fisher Scientific, PHC7045), 25 ng ml^−1^ β-NGF (Thermo Fisher Sceintific, PHG0126), 25 ng ml^−1^ NT-3 (Thermo Fisher Sceintific, PHC7036) and CEPT cocktail^46^. After 24 h, CEPT cocktail was removed and replaced with 1 µM PD0332991 (Tocris, cat. no. 4786/10). Half-medium changes were performed every 3 days for 14 further days of neuronal maturation. On day 7, after the onset of neuronal maturation, cells were transduced with a lentiviral vector at a multiplicity of infection of 5 for 24 h before complete medium change was performed.

### Human iPS cell sensory neuron treatment and imaging analysis

On day 14 of neuronal maturation, cells were treated with TSP-1 (RnD systems 3074-TH) at varying concentrations for 10 min. Cells were then imaged at 20× magnification in FITC channel at 150 ms exposure using ImageXpress Micro Confocal (Molecular Devices) before the addition of 10 µM FSK (Tocris 1099) (pre-image). Cells were again imaged immediately after the application of FSK (post-image). All images were subjected to background removal in Fiji ImageJ software. Cell bodies were identified, and their intensity in pre- and post-FSK treatment images was measured using Cell Profiler software^47^. FC in total cell body intensity was calculated for each well.

### Data and statistical analysis

Statistical analysis, including animal numbers (*n*) and *P* values, are included in the figure legends. Statistical analysis was performed using GraphPad Prism 9. Data distribution was assumed to be normal, but this was not formally tested. All single-cell sequencing analysis was performed using R version 4.2.3. The web resource used to present our data (http://painseq.shinyapps.io/immune/) was built using R shiny apps and Shiny Cell v2.1 (ref. ^48^).

### Reporting summary

Further information on research design is available in the Nature Portfolio Reporting Summary linked to this article.

## Online content

Any methods, additional references, Nature Portfolio reporting summaries, source data, extended data, supplementary information, acknowledgements, peer review information; details of author contributions and competing interests; and statements of data and code availability are available at 10.1038/s41590-024-01857-2.

### Supplementary information

Reporting Summary

Supplementary Data File 1 List of receptor genes used for INDRA interactome.

Supplementary Data File 2 List of ligand genes used for INDRA interactome.

Supplementary Data File 3 List of enzyme genes used for INDRA interactome.

Supplementary Data File 4 List of ion channel genes used for INDRA interactome.

Supplementary Data File 5 List of enzyme–product relations.

Supplementary Data File 6 Output of INDRA cell–cell interactome used as input for CellChat analysis.

Supplementary Data File 7 Immune-DRG neurons interactome in zymosan at *T*_max_.

Supplementary Data File 8 Immune-DRG neurons interactome in incision at *T*_max_.

Supplementary Data File 9 Immune-DRG neurons interactome in UV burn at *T*_max_.

Supplementary Tables 1–5 Supplementary Tables 1–5.

## Data Availability

Raw immune cell scRNA-seq files from healthy and injured skin samples will be deposited at the Gene Expression Omnibus repository (GEO) with a GEO accession number (GSE255686). The analyzed dataset from immune cell scRNA-seq is also available at http://painseq.shinyapps.io/immune/.

## Code availability

Source code to generate the interactome and the interactome gene lists used in this study is available at https://github.com/gyorilab/neuroimmune_interactome.
