# Supplementary material for: Nociceptor-immune interactomes reveal insult-specific immune signatures of pain
Source: Nat Immunol. 2024 May 28;25(7):1296–305. doi: 10.1038/s41590-024-01857-2 (PMC11224023; doi:10.1038/s41590-024-01857-2)
Supplement: Supplementary file 1 — Reporting Summary [file 41590_2024_1857_MOESM1_ESM.pdf]

nature portfolio

Corresponding author(s): Clifford Woolf
Last updated by author(s): Apr 22, 2024

Reporting Summary

Nature Portfolio wishes to improve the reproducibility of the work that we publish. This form provides structure for consistency and transparency in reporting. For further information on Nature Portfolio policies, see our Editorial Policies and the Editorial Policy Checklist.

Statistics

For all statistical analyses, confirm that the following items are present in the figure legend, table legend, main text, or Methods section.

n/a	Confirmed	
☐	☒	The exact sample size (*n*) for each experimental group/condition, given as a discrete number and unit of measurement
☐	☒	A statement on whether measurements were taken from distinct samples or whether the same sample was measured repeatedly
☐	☒	The statistical test(s) used AND whether they are one- or two-sided<br>*Only common tests should be described solely by name; describe more complex techniques in the Methods section.*
☒	☐	A description of all covariates tested
☒	☐	A description of any assumptions or corrections, such as tests of normality and adjustment for multiple comparisons
☐	☒	A full description of the statistical parameters including central tendency (e.g. means) or other basic estimates (e.g. regression coefficient) AND variation (e.g. standard deviation) or associated estimates of uncertainty (e.g. confidence intervals)
☐	☒	For null hypothesis testing, the test statistic (e.g. *F*, *t*, *r*) with confidence intervals, effect sizes, degrees of freedom and *P* value noted<br>*Give P values as exact values whenever suitable.*
☒	☐	For Bayesian analysis, information on the choice of priors and Markov chain Monte Carlo settings
☒	☐	For hierarchical and complex designs, identification of the appropriate level for tests and full reporting of outcomes
☐	☒	Estimates of effect sizes (e.g. Cohen's *d*, Pearson's *r*), indicating how they were calculated

Our web collection on statistics for biologists contains articles on many of the points above.

Software and code

Policy information about availability of computer code

Data collection	Source code to generate the interactome and the interactome gene lists used in this study are available at https://github.com/gyorilab/neuroimmune_interactome.
Data analysis	Statistical analysis, including animal numbers (n) and p values, are included in the figure legends. Statistical analysis was performed using Graphpad Prism 9. All single-cell sequencing analysis was performed using R version 4.2.3. The web resource used to present our data http://painseq.shinyapps.io/immune/ was built using R shiny apps and Shiny Cell42.

For manuscripts utilizing custom algorithms or software that are central to the research but not yet described in published literature, software must be made available to editors and reviewers. We strongly encourage code deposition in a community repository (e.g. GitHub). See the Nature Portfolio guidelines for submitting code & software for further information.

Data

Policy information about availability of data

All manuscripts must include a data availability statement. This statement should provide the following information, where applicable:

- Accession codes, unique identifiers, or web links for publicly available datasets
- A description of any restrictions on data availability
- For clinical datasets or third party data, please ensure that the statement adheres to our policy

Raw immune cell scRNA-seq files from healthy and injured skin samples will be deposited at the Gene Expression Omnibus repository (GEO) with a GEO accession

number (GSE255686). The analyzed dataset from immune cell scRNA seq is also available on http://painseq.shinyapps.io/immune/. Source code to generate the interactome and the interactome gene lists used in this study is available at: https://github.com/gyorilab/neuroimmune_interactome.

Research involving human participants, their data, or biological material

Policy information about studies with human participants or human data. See also policy information about sex, gender (identity/presentation), and sexual orientation and race, ethnicity and racism.

Reporting on sex and gender	N/A
Reporting on race, ethnicity, or other socially relevant groupings	N/A
Population characteristics	N/A
Recruitment	N/A
Ethics oversight	N/A

Note that full information on the approval of the study protocol must also be provided in the manuscript.

Field-specific reporting

Please select the one below that is the best fit for your research. If you are not sure, read the appropriate sections before making your selection.

☒ Life sciences ☐ Behavioural & social sciences ☐ Ecological, evolutionary & environmental sciences

For a reference copy of the document with all sections, see nature.com/documents/nr-reporting-summary-flat.pdf

Life sciences study design

All studies must disclose on these points even when the disclosure is negative.

Sample size	Sample size for behavioral experiments was based on previous data in the lab. Sample size for scRNA seq experiment was based on what is established in the field at n=2 for single cell transcriptomics. No statistical methods were used to pre-determine sample sizes but our sample sizes are similar to those reported in previous publications (zhang et. al., 2022).
Data exclusions	No data was excluded
Replication	All attempts at replication were successful. The replication number for each experiment is included in the legends.
Randomization	For behavior experiments, mice were chosen randomly from each cage. For Ca imaging experiments, dishes with DRG cultures were chosen randomly for treatment groups.
Blinding	For behavior experiments, person inducing the stimulus was different from the person investigating the behavior who was fully blinded.

Reporting for specific materials, systems and methods

We require information from authors about some types of materials, experimental systems and methods used in many studies. Here, indicate whether each material, system or method listed is relevant to your study. If you are not sure if a list item applies to your research, read the appropriate section before selecting a response.

Materials & experimental systems

n/a	Involved in the study	
☐	☒	Antibodies
☐	☒	Eukaryotic cell lines
☒	☐	Palaeontology and archaeology
☐	☒	Animals and other organisms
☒	☐	Clinical data
☒	☐	Dual use research of concern
☒	☐	Plants

Methods

n/a	Involved in the study	
☒	☐	ChIP-seq
☐	☒	Flow cytometry
☒	☐	MRI-based neuroimaging

Antibodies

Antibodies used	All antibodies used in this study were specific to mice. Flow cytometry antibodies include CD45-FITC (1:400, Biolegend, Cat no.103107), CD64-PE-594 (1:600, Biolegend, Cat no. 139319), CD11C-APC (1:400, Biolegend, 101211) Cd11b- eFluor 780 (1:400, ebioscience, 47-0112-82), Ly6G-PE(1:800, Biolegend, 127607) and Ly6c-BV711 (1:2000, Bioelgend, 128037). IHC antibodies include (Rb anti-PGP9.5 abcam ab108986 1:500, Goat anti-CD47 RnD Systems AF1866 1:200), (Donkey anti-Rb Cy3 Jackson ImmunoResearch 711-165-152 1:500, Donkey anti-Goat 647 Thermo Fischer A-21447 1:500).
Validation	All antibodies have been validated by manufacturer.

Eukaryotic cell lines

Policy information about cell lines and Sex and Gender in Research

Cell line source(s)	IPSC lines were obtained from Lonza iPS (LiPS.GR-1-1). This cell line was derived from a male donor.
Authentication	No authentication was performed.
Mycoplasma contamination	No mycoplasma contamination was detected.
Commonly misidentified lines (See ICLAC register)	N/A

Animals and other research organisms

Policy information about studies involving animals; ARRIVE guidelines recommended for reporting animal research, and Sex and Gender in Research

Laboratory animals	Both Male and Female mice were used in all the behavior experiments. Single-cell RNA sequencing was performed on 8 wks old female mice. 8-12-week-old C57BL/6J mice were obtained from the Jackson Laboratory (JAX:000664) as were Cd47 deficient mice (Jax:003173). All mice in this study were kept on a 12h light cycle, at 21–23°C, with 30–50% humidity.
Wild animals	No Wild animals were used.
Reporting on sex	All the information on sex used in individual experiments in included in the text.
Field-collected samples	Please state here that no field collected samples were used in the study.
Ethics oversight	All animal experiments were conducted according to institutional animal care and safety guidelines at Boston Children's Hospital and Harvard Medical School

Note that full information on the approval of the study protocol must also be provided in the manuscript.

Flow Cytometry

Plots

Confirm that:

☒ The axis labels state the marker and fluorochrome used (e.g. CD4-FITC).

☒ The axis scales are clearly visible. Include numbers along axes only for bottom left plot of group (a 'group' is an analysis of identical markers).

☒ All plots are contour plots with outliers or pseudocolor plots.

☒ A numerical value for number of cells or percentage (with statistics) is provided.

Methodology

Sample preparation	The planter skin of the mouse hind paw was dissected, separating the muscle and collected into 1% BSA containing RPMI. The skin was minced using scissors into 1-2mm pieces. Liberase TM (Roche) was added to the media at a final concentration of 0.5 mg/ml. Tissue was digested at 37c while vortexing at 1000rpm for 90 min.
Instrument	The digested tissue was strained using a 100uM strainer to obtain a single-cell suspension used for flow sorting on BD AriaII and single cell transcriptomics.
Software	Analysis software FlowJo 10
Cell population abundance	Cell abundance is shown in Supp Fig 2 and quantified in Fig 1F. Purity was detemrined by postsort anlaysis. The post sort data is not included since these cells were used for single cell RNA seq for which non-CD45+ populations are indicated.

Gating strategy	Gating strategy for immune cell sorting is showin in Supp Fig 1A. Gating strategy for myeloid cells is shown in Supp Fig 2A.

☒ Tick this box to confirm that a figure exemplifying the gating strategy is provided in the Supplementary Information.
